# Saikosaponin a inhibits LPS-induced inflammatory response by inducing liver X receptor alpha activation in primary mouse macrophages

**DOI:** 10.18632/oncotarget.9863

**Published:** 2016-06-06

**Authors:** Zhengkai Wei, Jingjing Wang, Mingyu Shi, Weijian Liu, Zhengtao Yang, Yunhe Fu

**Affiliations:** ^1^ Department of Clinical Veterinary Medicine, College of Veterinary Medicine, Jilin University, Changchun, Jilin Province, People's Republic of China

**Keywords:** saikosaponin a, NF-κB, IRF3, TLR4, lipid raft, Immunology and Microbiology Section, Immune response, Immunity

## Abstract

The aim of this study was to investigate the effects of SSa on LPS-induced endotoxemia in mice and clarify the possible mechanism. An LPS-induced endotoxemia mouse model was used to confirm the anti-inflammatory activity of SSa *in vivo*. The primary mouse macrophages were used to investigate the molecular mechanism and targets of SSa *in vitro*. *In vivo*, the results showed that SSa improved survival during lethal endotoxemia. *In vitro*, our results showed that SSa dose-dependently inhibited the expression of TNF-α, IL-6, IL-1β, IFN-β-and RANTES in LPS-stimulated primary mouse macrophages. Western blot analysis showed that SSa suppressed LPS-induced NF-κB and IRF3 activation. Furthermore, SSa disrupted the formation of lipid rafts by depleting cholesterol and inhibited TLR4 translocation into lipid rafts. Moreover, SSa activated LXRα, ABCA1 and ABCG1. Silencing LXRα abrogated the effect of SSa. In conclusion, the anti-inflammatory effects of SSa is associated with activating LXRα dependent cholesterol efflux pathway which result in disrupting lipid rafts by depleting cholesterol and reducing translocation of TLR4 to lipid rafts, thereby attenuating LPS mediated inflammatory response.

## INTRODUCTION

Sepsis is a systemic inflammatory response syndrome that often results from the stimulation of pathogen components [1]. It is characterized by overproduction of inflammatory cytokines and leads to the lethal multiple organ damage. LPS is one of the most important factors that lead to sepsis. LPS has been reported to activate TLR4 signaling pathway, which subsequently induced the production of inflammatory mediators [2, 3]. Toll-like receptors (TLRs) are a large family of Type I transmembrane receptors that play an integral role in the innate immune system. There have been reported 13 kinds of recently [4-6]. TLR4 is activated by LPS, the integral molecules within the outer membrane of gram-negative bacteria [7-9]. Upon stimulation by LPS, TLR4 is recruited to lipid rafts and interacts with its adaptor molecules, leading to activation of MyD88-dependent and MyD88-independent signaling pathways, resulting in NF-κB and IRF3 activation and cytokines production.

The liver X receptors α and β (LXRα and LXRβ) are members of the nuclear receptor family of proteins that are critical for the control of lipid homeostasis in vertebrates [10]. Activation of LXR induces expression of genes involved in cholesterol efflux such as ABCA1 and ABCG1 [11]. ABCA1 is a lipid pump that effluxes cholesterol and phospholipid out of cells [12, 13]. Activation of ABCA1 and ABCG1 could induce cholesterol efflux from plasma membrane microdomains known as lipid rafts [14]. Lipid rafts are microdomains of the plasma membrane which are enriched in cholesterol and sphingolipids. They serve as a platform for signal transduction and play an important role in TLR4 signal pathway [15]. Treatment with raft-disrupting drugs could inhibit the LPS-induced NF-κB activation and TNF-α production [16, 17].

**Figure 1:**
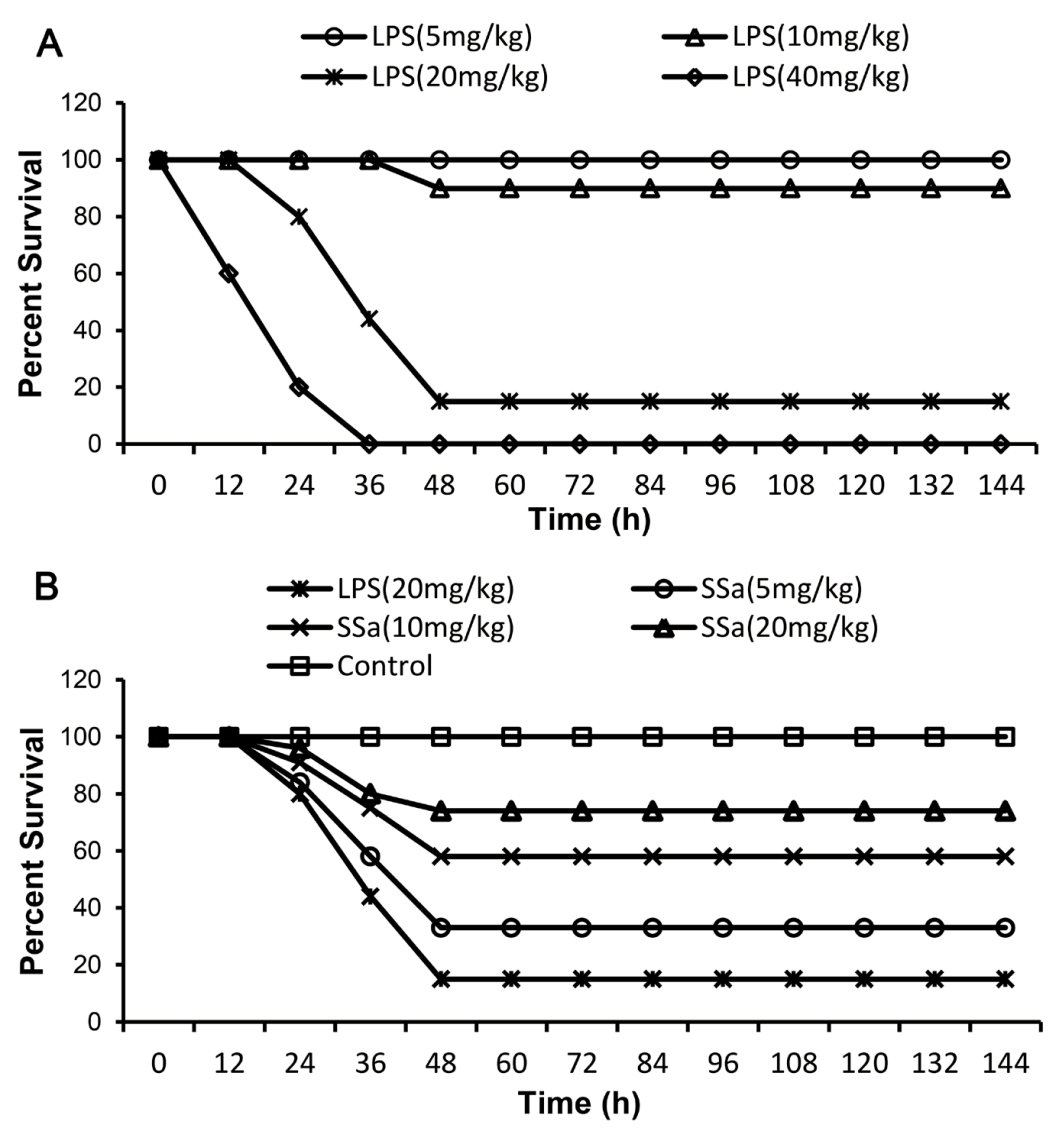
Effects of SSa on LPS-induced lethality in mice Mice were given an intraperitoneal injection of SSa (5, 10 and 20 mg/kg) 1 h prior to LPS challenged. **A.** The survival rate of mice challenged with LPS of different doses. **B.** Effect of SSa (5, 10 and 20 mg/kg) treatment on LPS-induced lethality. The survival was monitored every 12 hour for 7 days. #*P* < 0.01 *vs*. control group, *P < 0.05 and **P < 0.01 *vs*. LPS group.

Saikosaponin a (Figure 1), the major triterpenoid saponin derivatives from Radix bupleuri (RB), is responsible for the plant's pharmacological activities. It has been shown that SSa exhibited a board spectrum anti-inflammatory effects [18]. SSa was found to inhibit inflammatory mediators including iNOS, TNF-α and IL-1β production in LPS-stimulated RAW264.7 cells [19]. It has been reported that SSa has specific inhibitory effects on NF-κB activation [20]. However, the molecular targets of the anti-inflammatory actions of SSa has not been clearly elucidated. The aim of this study was to investigate the effect of SSa on LPS-induced endotoxemia in mice and to identify the molecular targets of SSa in the TLR4 signaling pathway.

## RESULTS

### Effects of SSa on LPS-mediated mortality

To determine a suitable concentration of LPS for inducing endotoxemia, the mice were challenged with LPS (5-40 mg/kg), the dose response of LPS on mortality was shown in Figure 1A. Mice were given 5, 10, 20, 40 mg/kg of LPS, the mortality rates were 0 %, 10 %, 85 %, and 100 %, respectively. Therefore, 20 mg/kg LPS was chosed as lethal dosage to induce endotoxemia in mice. The effect of SSa on LPS-induced mortality was assessed by measuring survival of mice challenged with 20 mg/kg of LPS. As shown in Figure 1B, mice receiving 5, 10 or 20 mg/kg SSa were 33 %, 58 % and 74 % protective respectively.

### Effects of SSa on cell viability

**Figure 2:**
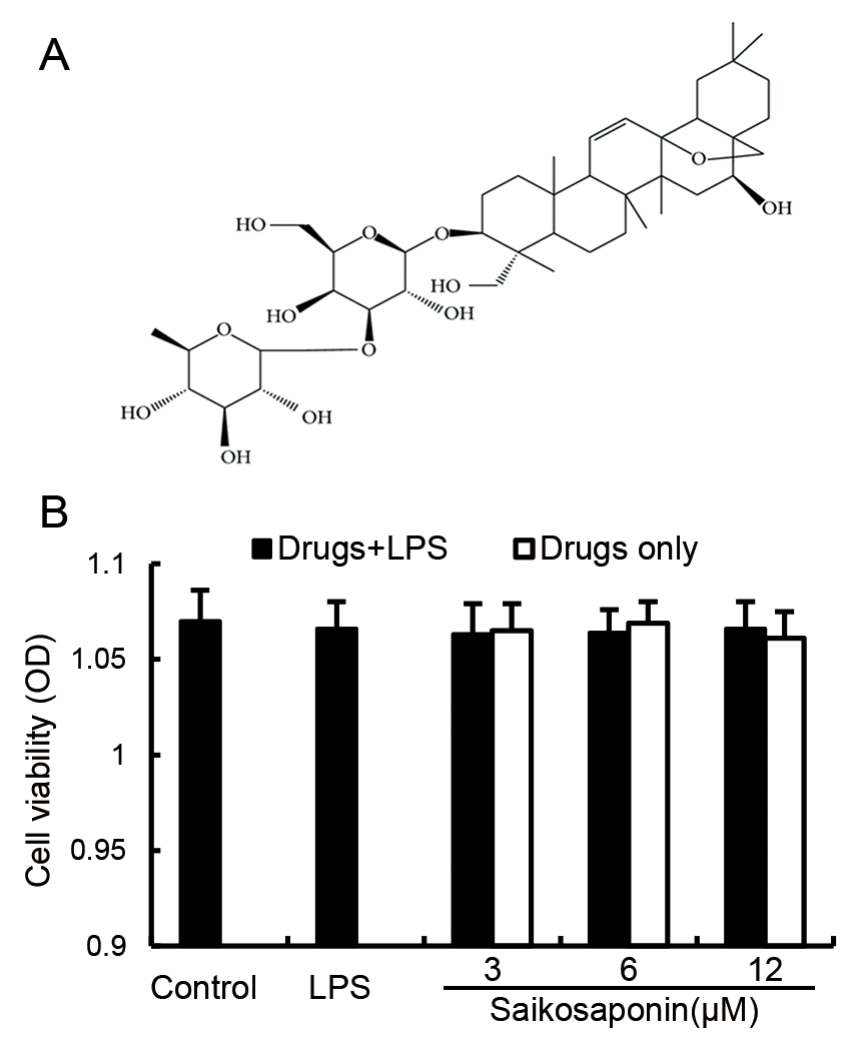
**A.** Chemical structure of SSa. **B.** Effect of SSa on the cell viability of primary mouse macrophages. Cells were cultured with different concentrations of SSa (0-100μM) 1h, followed by stimulation with 50μl LPS. After 18h of LPS stimulation, 20μl MTT (5mg/ml) was added to each well, and the cells were further incubated for an additional 4 h. The supernatant was removed and the formation of formazan was resolved with 150μl/well of DMSO. The cell viability was determined by MTT assay. The values presented are the means ± SEM of six independent experiments.

The potential cytotoxicity of SSa was evaluated by the MTT assay after incubating cells for 18 h in the absence or presence of LPS, the result showed that cell viabilities were not affected by the SSa at concentrations used (3, 6, 12 μM) (Figure 2B). Thus, the effects of SSa on primary mouse macrophages were not attributable to cytotoxic effects.

### SSa dose-dependently inhibits the secretion of cytokines in LPS-stimulated primary mouse macrophages

**Figure 3:**
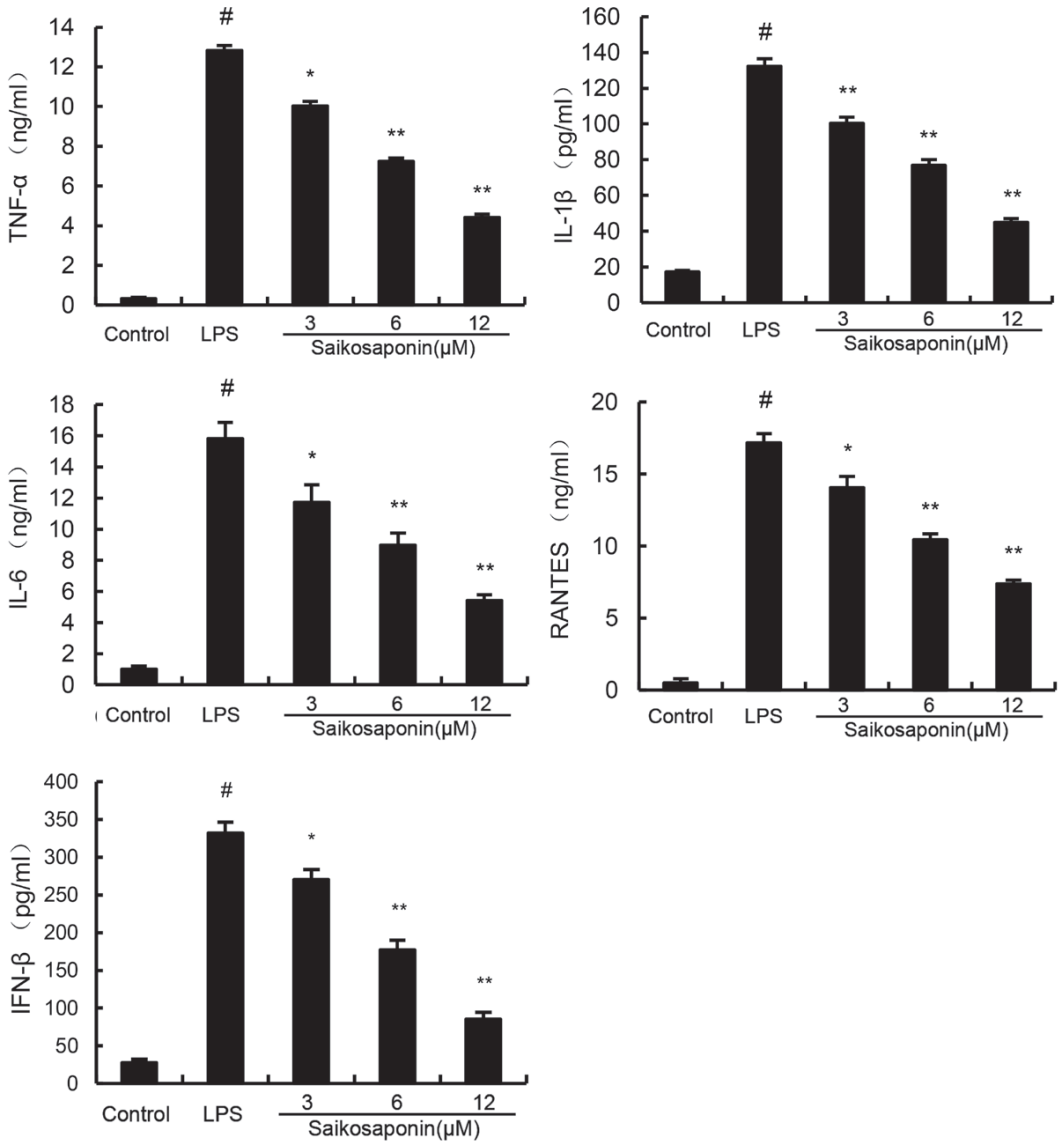
SSa inhibits lipopolysaccharide (LPS)-induced cytokine production in a dose-dependent manner Cells were treated with 0.1 μg/mL LPS in absence or presence of SSa (3, 6, 12 μM) for 6 h. Levels of TNF-α, IL-1β, IL-6, IFN-β, and RANTES in culture supernatants were measured by ELISA. The data presented are the means ± SEM of six independent experiments and differences between mean values were assessed by Students's *t*-test. #*P* < 0.05 *vs*. control group; *P < 0.05, **P < 0.01 *vs*. LPS group.

To analyze the potential anti-inflammatory effects of SSa, we determined whether SSa affected the expression of cytokines in LPS-stimulated primary mouse macrophages. The expression of TNF-α, IL-6, IL-1β, IFN-β, and RANTES were detected by ELISA. The results showed that SSa suppressed TNF-α, IL-6, IL-1β, IFN-β, and RANTES expression in LPS-stimulated primary mouse macrophages in a dose-dependent manner (Figure 3).

### SSa suppresses LPS-induced NF-κB and IRF3 activation

**Figure 4:**
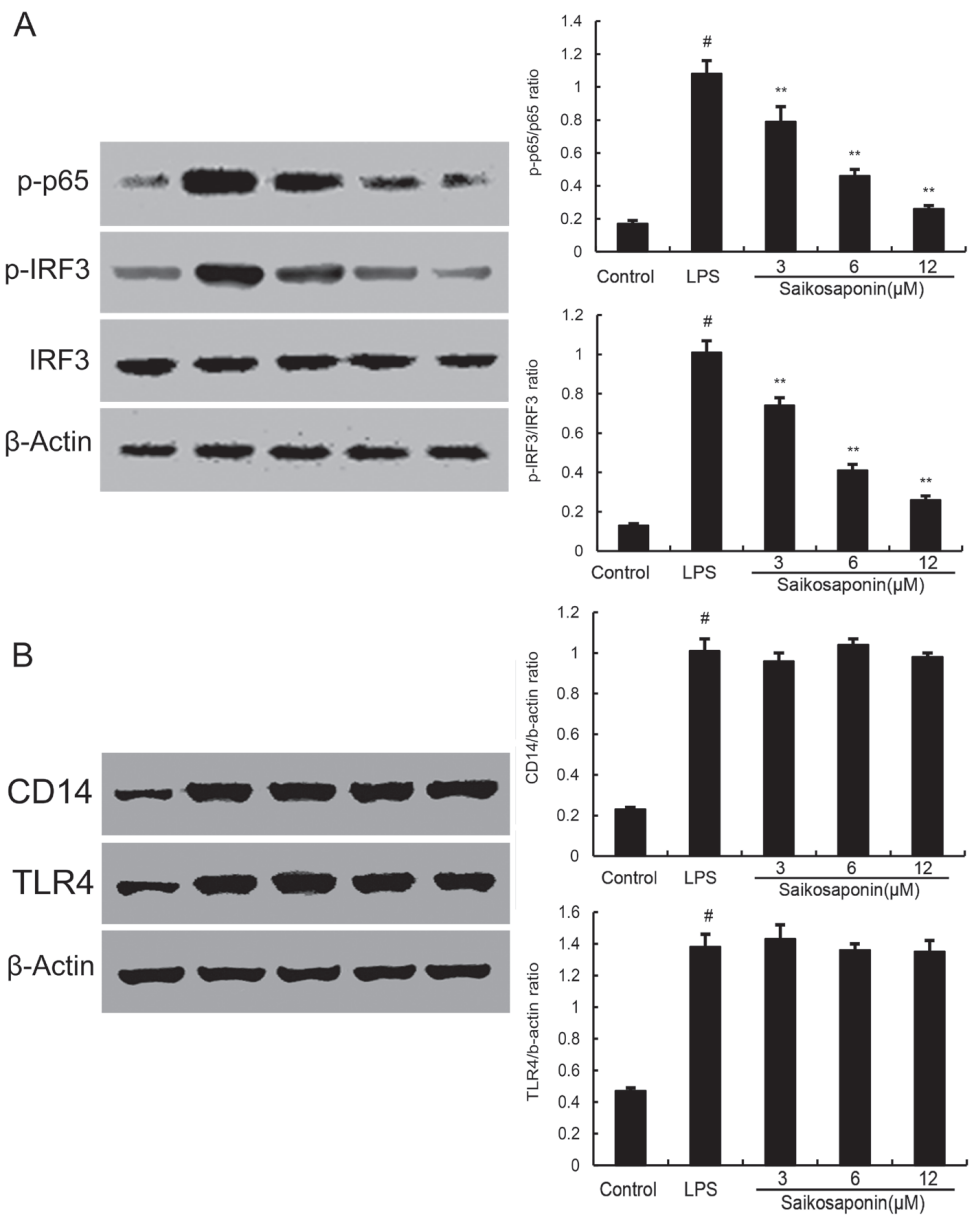
**A.** SSa inhibits lipopolysaccharide (LPS)-induced NF-κB and IRF3 activation. Cells were preincubated with SSa (3, 6, 12μM) for 12 h and then treated with 0.1μg/mL LPS for 1h. B. Effects of SSa on lipopolysaccharide (LPS)-induced TLR4 and CD14 expression. Cells were preincubated with SSa (3, 6, 12μM) for 12 h and then treated with 0.1μg/mL LPS for 3h. Protein samples were analyzed by western blot with specific antibodies. β-actin was used as a control. The data presented are the means ± SEM of six independent experiments and differences between mean values were assessed by Students's *t*-test. #*P* < 0.05 *vs*. control group; *P < 0.05, **P < 0.01 *vs*. LPS group.

It is well-known that NF-κB and IRF3 are important signaling molecules in the development of inflammatory diseases. Activation of TLR4 induces two signaling pathways: MyD88 and TRIF dependent signaling pathways which induces NF-κB and IRF3 activation and finally results in the release of inflammatory cytokines. To test whether the inhibition of inflammatory response by SSa is mediated through the NF-κB and IRF3 pathway, NF-κB and IRF3 protein were determined by Western blotting. The results showed that SSa significantly inhibit the activation of NF-κB and IRF3 (Figure 4A).

### Effect of SSa on the expression of TLR4 and CD14

To test whether the inhibition of inflammatory response by SSa exerted through inhibiting TLR4 and CD14 expression, we detected TLR4 and CD14 expression by Western blotting. As shown in Figure 4B, the results showed that SSa did not inhibit TLR4 and CD14 expression induced by LPS.

### SSa inhibits translocation of TLR4 to lipid rafts

**Figure 5:**
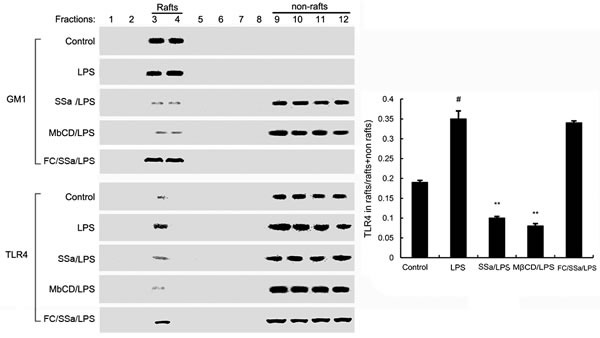
Recruitment of TLR4 into lipid rafts by SSa Primary mouse macrophages were pretreated with SSa or MβCD, then treated with 0.1μg/mL LPS. The cells were lysed and subjected to discontinuous sucrose density gradient centrifugation as described in Materials and methods. Fractions were analyzed using CTxB conjugated to horseradish peroxidase (GM1) or anti-TLR4 primary antibody. Fractions 3-4 correspond to lipid rafts. Fractions 9-12 correspond to non-lipid rafts. TLR4 content of macrophage lipid rafts was calculated as a percentage of total membrane TLR4 (lipid rafts + nonrafts). The values presented are the means ± SEM of six independent experiments and differences between mean values were assessed by Students's *t*-test. #*P* < 0.05 *vs*. control group; *P < 0.05, **P < 0.01 *vs*. LPS group.

Lipid rafts are involved with TLR4 signaling. Stimulating cells with LPS can induce TLR4 recruit to lipid rafts. To further address the potential anti-inflammatory effects of SSa, we determined the effects of SSa on the translocation of TLR4 to lipid rafts. We isolated raft fractions and examined the translocation of TLR4 by western blotting. The results showed that LPS stimulation induced localization of TLR4 to raft fractions. This effect was prevented by pretreatment with SSa or MβCD (Figure 5).

### SSa disrupts the formation of lipid rafts in cell membranes by depleting cholesterol

**Figure 6:**
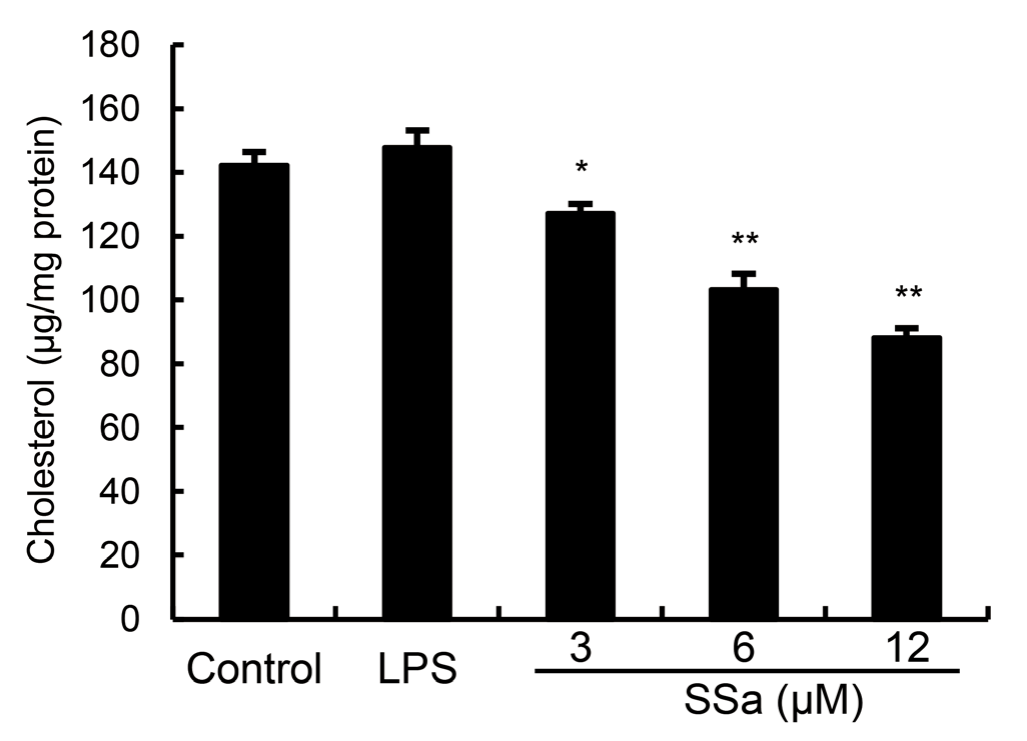
Effects of SSa on membrane lipid rafts cholesterol levels Primary mouse macrophages were treated with SSa (3, 6, 12μM) for 12 h. Membrane cholesterol levels were measured by gas-liquid chromatography and the results were plotted as μg cholesterol/mg protein. The values presented are the means ± SEM of six independent experiments and differences between mean values were assessed by Students's *t*-test (*P < 0.05, **P < 0.01).

In this study, we detected whether SSa exerts an anti-inflammatory property by disrupting lipid rafts. Cholesterol level of lipid raft was assayed by gas-liquid chromatography. The results showed that SSa disrupted the lipid rafts by removing of cholesterol from lipid rafts in a dose-dependent manner (Figure 6).

### Cholesterol replenishment prevents the anti-inflammatory effect of SSa

**Figure 7:**
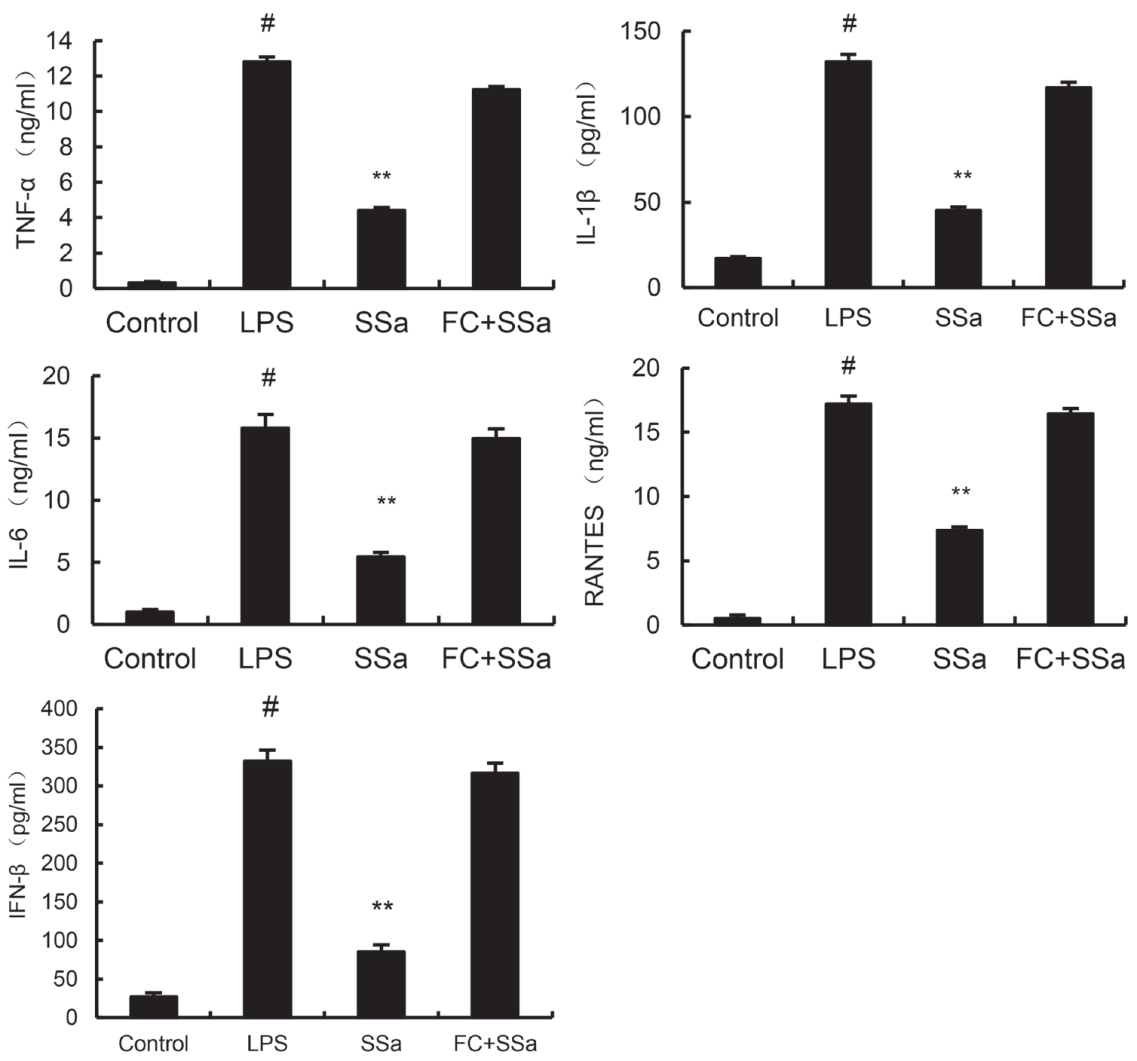
Cholesterol replenishment prevents the anti-inflammatory effect of SSa Primary mouse macrophages were treated with SSa (3, 6, 12μM) or MβCD (10mM) at 37°C for 60min. Subsequently the cells were washed with PBS and incubated with medium alone or medium containing water-soluble cholesterol (84μg/mL) for 30min. Cells were treated with 0.1 μg/mL LPS for 6 h. Levels TNF-α, IL-6, IL-1β, IFNβ and RANTES in culture supernatants were measured by ELISA. The data presented are the means ± SEM of six independent experiments and differences between mean values were assessed by ANOVA. #*P* < 0.05 *vs*. control group; *P < 0.05, **P < 0.01 *vs*. LPS group.

To further investigate the anti-inflammatory mechanism of SSa, cholesterol replenishment experiments were carried out. As shown in Figure 4, the inhibition effect of SSa on LPS-induced TLR4 translocation to lipid rafts were abolished. Meanwhile, the inhibitory effect of SSa on LPS-induced inflammatory cytokines were also abolished (Figure 7).

### SSa up-regulates the expression of LXRα, ABCA1 and ABCG1 in primary mouse macrophages

**Figure 8:**
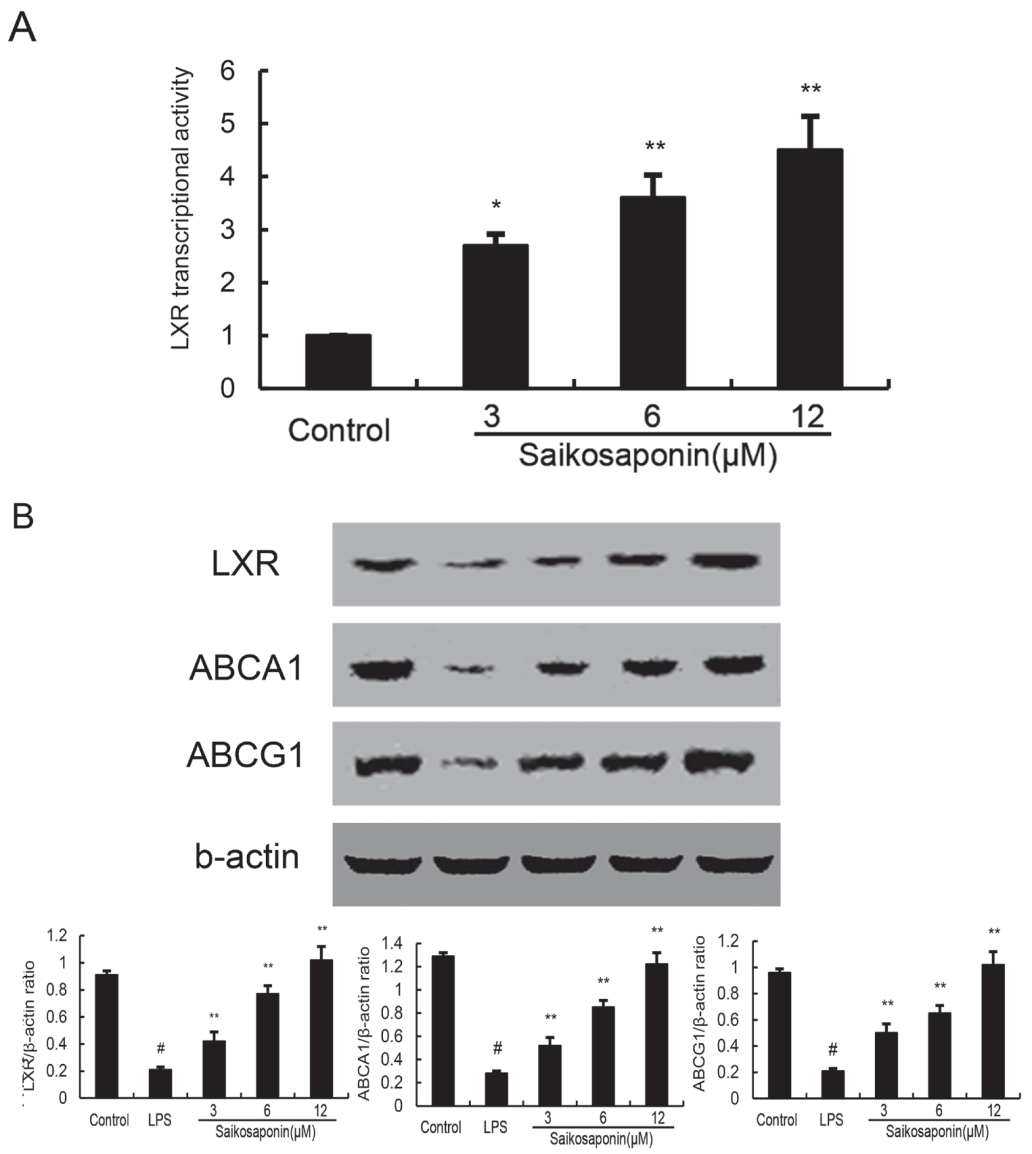
Effects of SSa on LXR transcriptional activity and LXRα, ABCA1 expression **A.** Cells were transfected with LXRE-driven luciferase reporter vector (LXRE-tk-Luc) and β-galactosidase control vector (Promega). Six hour later, cells were treated with SSa for 12h. Relative luciferase activity was determined by normalization with β-galactosidase activity (*P < 0.05, **P < 0.01). **B.** effects of SSa on LXRα and ABCA1 expression. Cells were SSa (3, 6, 12μM) for 12h. Protein samples were analyzed by western blot with specific antibodies. β-actin was used as a control. The values presented are the means ± SEM of six independent experiments and differences between mean values were assessed by Students's *t*-test (*P < 0.05, **P < 0.01).

LXRα play an important role in cholesterol homeostasis by regulatory sensors of cholesterol levels in cells. Activation of LXRα induces expression of genes involved in cholesterol efflux such as ABCA1 and ABCG1. In this study, we performed a luciferase reporter gene assay to test whether SSa could enhance transcriptional activity of LXRα. As shown in Figure 8A, SSa dose-dependently increased expression of the LXR luciferase reporter gene. Meanwhile, the expression of LXRα, ABCA1 and ABCG1 were detected by Western blotting. As shown in Figure 8B, LPS inhibited the expression of LXRα, ABCA1 and ABCG1. SSa up-regulated the expression of LXRα, ABCA1 and ABCG1 in a dose manner.

### Knockdown of LXRα abrogated the effects of SSa on ABCA1 expression, membrane cholesterol levels, and LPS induces inflammatory response in primary mouse macrophages

**Figure 9:**
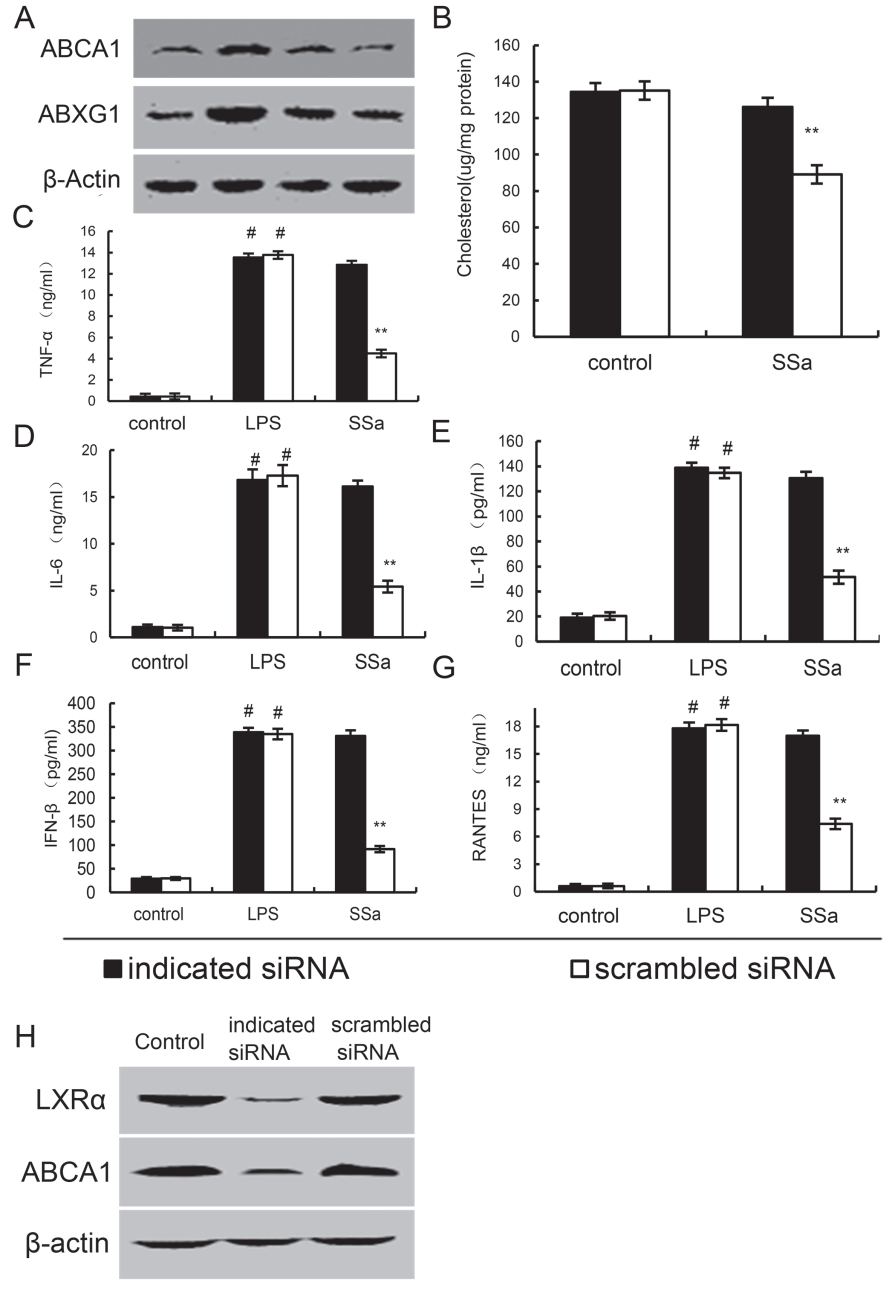
Knockdown of LXRα abrogated the effects of SSa on ABCA1, ABCG1 expression, membrane cholesterol levels, and LPS induces inflammatory response in primary mouse macrophages Primary mouse macrophages were transfected with a siRNA specific for LXRα, or a scrambled siRNA (negative control) as indicated. Then the cells were treated with SSa (12μM) for 12h. The ABCA1 and ABCG1 expression **A.** and membrane cholesterol levels **B.** were detected. Meanwhile, the cells were treated with SSa (12μM) for 12h and stimulated by 0.1μg/mL LPS for 6 h. Levels of TNF-α **C.**, IL-6 **D.**, IL-1β **E.**, IFNβ **F.** and RANTES **G.** in culture supernatants were measured by ELISA. **H.** Effects of siRNA on LXRα and ABCA1 expression. The data presented are the means ± SEM of six independent experiments and differences between mean values were assessed by Students's *t*-test. #*P* < 0.05 *vs*. control group; *P < 0.05, **P < 0.01 *vs*. LPS group.

**Figure 10:**
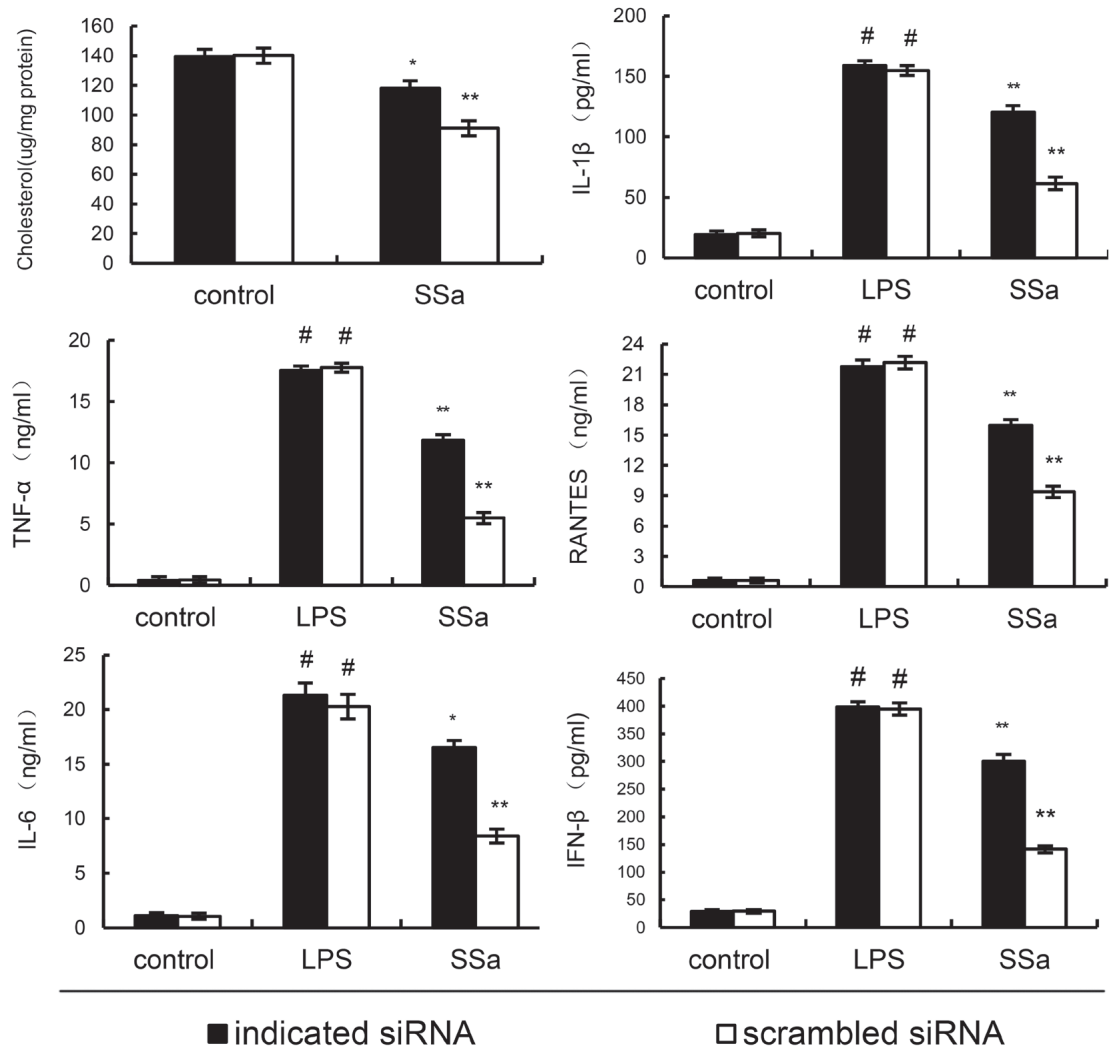
Knockdown of ABCA1 partly abrogated the effects of SSa membrane cholesterol levels, and LPS induces inflammatory response in primary mouse macrophages Primary mouse macrophages were transfected with a siRNA specific for ABCA1, or a scrambled siRNA (negative control) as indicated. Then the cells were treated with SSa (12μM) for 12h. Membrane cholesterol levels were detected. Meanwhile, the cells were treated with SSa (12μM) for 12h and stimulated by 0.1μg/mL LPS for 6 h. Levels of TNF-α, IL-6, IL-1β, IFN-β and RANTES in culture supernatants were measured by ELISA. The data presented are the means ± SEM of six independent experiments and differences between mean values were assessed by Students's *t*-test. #*P* < 0.05 *vs*. control group; *P < 0.05, **P < 0.01 *vs*. LPS group.

To detect whether the anti-inflammatory effects of SSa is LXRα dependent, LXRα was silencing in primary mouse macrophages by their specific siRNA. When LXRα was silenced, the effects of SSa on ABCA1, ABCG1 expression, membrane cholesterol levels, the expression of cytokines induced by LPS were reversed (Figure 9). Meanwhile, we found that knockdown of ABCA1, the effects of SSa on membrane cholesterol levels, the expression of cytokines induced by LPS were partly reversed (Figure 10).

## DISCUSSION

Although the anti-inflammatory activity of SSa are widely described, a detailed analysis of its molecular targets remains unclear to date. In the present study, we evaluated the effects of SSa on LPS-induced endotoxemia in mice and clarify the possible mechanism. Our results demonstrated that SSa exerts an anti-inflammatory property by disrupting and removing of cholesterol from lipid rafts and inhibiting translocation of TLR4 to lipid rafts, thereby attenuating LPS mediated NF-κB and IRF3 activation and inflammatory cytokines production. These effects of SSa were regulated by activating LXR-ABCA1-dependent cholesterol efflux. Knockdown of LXRα abrogated the anti-inflammatory effects of SSa.

The cytokines play an important role in inflammatory diseases [21, 22]. LPS activates the TLR4-mediated signaling pathway and leads to the activation of NF-κB and IRF3 to regulate the release of cytokines such as TNF-α, IL-1β, IL-6 and RANTES [23, 24]. To explore the potential anti-inflammatory effects of SSa *in vitro*, the effects of SSa on NF-κB and IRF3 activation and the production of these cytokines were examined. The results showed that SSa pre-treatment significantly inhibit LPS-induced NF-κB and IRF3 activation and the production of TNF-α, IL-1β, IL-6 and RANTES in primary mouse macrophages.

TLR4 is the major receptor for LPS. Upon stimulation by LPS, TLR4 is recruited to lipid rafts and subsequently interacts with its adaptor molecules, leading to activation of downstream targets [16]. Lipid rafts are plasma membrane microdomains that contain high concentrations of cholesterol and glycosphingolipids [25]. It is well known that lipid rafts provide platforms for the formation of receptor complexes and play fundamental roles in signaling transduction. Recently, some studies had shown that lipid rafts play an important role in LPS-induced signaling in macrophages [26]. In this study, our results demonstrated that SSa inhibited translocation of TLR4 to lipid rafts (Figure 4). Meanwhile, the results in Figure 5 showed that SSa disrupted the formation of lipid rafts by depleting cholesterol. Studies showed that treatment with raft-disrupting drugs (depleting cholesterol) could inhibit TLR4 translocation into lipid rafts and LPS induced NF-κB activation and TNF-α production [16, 17]. Meanwhile, our cholesterol replenishment results confirmed that cholesterol depletion contributed to the inhibition of LPS-induced inflammatory response by SSa. After cholesterol replenishment, TLR4 translocation to lipid raft increased in primary mouse macrophages treated with SSa (Figure 4). Overall, our results demonstrated that SSa disrupts lipid rafts by depleting cholesterol which leading to inhibition of TLR4 translocation to lipid raft and LPS-induced inflammatory responses.

The LXR nuclear receptors are intracellular sensors of cholesterol excess and are activated by various oxysterols [27]. LXRs regulate intracellular cholesterol levels through mediating the expression of ABCA1, which modulates cholesterol efflux and reverse cholesterol transport (RCT) from peripheral tissues. Reports have shown that macrophage ABCA1 inhibit TLR4 trafficking to lipid rafts by reduction of lipid rafts cholesterol [28]. To investigate the mechanism of SSa reducing lipid rafts cholesterol, the effects of SSa on LXRα and ABCA1 were detected. Our results showed that SSa increased LXRα and ABCA1 expression and decreased membrane cholesterol content. These results suggested that SSa activated LXRα-ABCA1 pathway by mediating cholesterol efflux to reduce lipid rafts cholesterol content in primary mouse macrophages.

To further confirm the involvement of the LXRα-ABCA1 pathway in the anti-inflammatory effect of SSa on primary mouse macrophages, LXRα was silenced by siRNA. We showed that when LXRα was silenced, the effects of SSa on ABCA1 expression, membrane cholesterol levels, the expression of cytokines induced by LPS were reversed. The present results obtained from LXRα knockdown support the critical role of the LXRα-ABCA1 pathway in the anti-inflammatory effects of SSa.

In conclusion, the studies demonstrate that SSa can inhibit the expression of TNF-α, IL-6, IL-1β and RANTES in LPS-stimulated macrophages. The promising anti-inflammatory effect of SSa on LPS-stimulated primary mouse macrophages is associated with up-regulation of the LXRα-ABCA1 pathway which result in disrupting lipid rafts and reducing translocation of TLR4 to lipid rafts, thereby suppressing TLR4 mediated NF-κB and IRF3 signaling pathways induced by LPS.

## MATERIALS AND METHODS

### Reagents

SSa (purity>98%) was purchased from the National Institute for the Control of Pharmaceutical and Biological Products (Beijing, China). Dimethyl sulfoxide (DMSO), LPS (Escherichia coli 055:B5), and 3-(4,5-dimethylthiazol-2-y1)-2,5-diphenyltetrazolium bromide (MTT) were purchased from Sigma Chemical Co. (St. Louis, MO, USA). Dulbecco's modified Eagle's medium (DMEM), Fetal bovine serum (FBS) were obtained from Hyclone. Mouse TNF-α, IL-6 and IL-1β enzyme-linked immunosorbent assay (ELISA) kits were purchased from Biolegend (CA, USA). Mouse RANTES ELISA kits were purchased from R&D Systems (Minneapolis, MN). Mouse mAb Phospho-NF-κB and mouse mAb NF-κB, Mouse mAb Phospho-IRF3 and rabbit mAb IRF3 were purchased from Cell Signaling Technology Inc (Beverly, MA). TLR4, LXRα, and ABCA1 antibodies were purchased from Santa Cruz Biotechnology. HRP-conjugated goat anti-rabbit antibodies were provided by GE Healthcare (Buckinghamshire, UK). All other chemicals were of reagent grade.

### Animals

Male BALB/c mice, 6-8 weeks, weighing approximately 18 to 20 g, were purchased from the Center of Experimental Animals of Baiqiuen Medical College of Jilin University (Jilin, China). And this study was approved by the Jilin University Animal Care and Use Committee. The protocols were reviewed and approved by the committee. The mice were housed in microisolator cages and received food and water. The laboratory temperature was 24 ± 1 °C, and relative humidity was 40-80%. Mice were housed for 4-6 days to adapt the environment before experimentation. All animal experiments were performed in accordance with the guide for the Care and Use of Laboratory Animals published by the US National Institutes of Health.

### LPS-induced endotoxemia in mice

The 48 healthy male BALB/c mice were randomly classified into four groups and challenged with LPS (5-40 mg/kg) by i.p. The mortality of mice was observed twice a day for 7 days. SSa was diluted with DMSO and further diluted with PBS. In drug testing, the effect of SSa (5, 10 and 20 mg/kg) on LPS-induced mortality was assessed by given SSa 1 h before LPS challenge. Survival in each group was assessed every 12 h for 7 days.

### Cell culture and treatment

Female C57 mice were injected i.p. with 2 ml of 4 % thioglycollate broth (Difco Laboratories, Detroit, MI). Four days later, peritoneal cells were harvested with phosphate-buffered saline (PBS). The cells were cultured in RPMI 1640 medium supplemented with 10 % FBS at 37 °C with 5 % CO_2_. Media was changed once every 48h. In all experiments, macrophages were incubated in the presence or absence of various concentrations of SSa that was always added 12 h prior to LPS (0.1μg/mL) treatment.

### MTT assay for cell viability

An MTT assay was used to measure cell viability. Briefly, primary mouse macrophages were plated at a density of 4×10^5^ cells/ml in 96-well plates in a 37 °C, 5% CO_2_ incubator for 1h, then the cells were treated with 50 μl of SSa at different concentrations (0-100μM) for 1h, followed by stimulation with 50 μl LPS. After 18 h of LPS stimulation, 20 μl MTT (5 mg/ml) was added to each well, and the cells were further incubated for an additional 4 h. The supernatant was removed and the formation of formazan was resolved with 150 μl/well of DMSO. The optical density was measured at 570 nm on a microplate reader (TECAN, Austria).

### ELISA assay

Primary mouse macrophages were seeded in 24-well plates (4×10^5^ cells/well), and incubated in the presence of either LPS alone 0.1 μg/ml, or LPS plus SSa (3, 6, 12 μM) for 6 h. Cell-free supernatants were subsequently employed for the cytokine assays using an ELISA kit, according to the manufacturer's instructions (BioLegend, USA).

### Western blot analysis

Primary mouse macrophages were seeded in 6-well plates and incubated for 24 h, then pretreated with SSa for 1 h. After LPS (0.1 μg/ml) stimulation for 1 h, the cells were collected and washed twice with cold PBS. Total proteins from cells were extracted by M-PER Mammalian Protein Extraction Reagent (Thermo). Protein concentration was determined through BCA method. The proteins (30 mg) were separated by SDS-PAGE using Tris-HCl Precast Gels and then transferred onto the PVDF membrane. The resulting membrane was blocked with phosphate buffer solution containing 0.05 % Tween-20 (PBS-T), supplemented with 3 % skim milk at room temperature for 2 h on a rotary shaker, and followed by PBS-T washing. The specific primary antibody diluted in PBS-T containing skim milk, was incubated with the membrane at 4 °C overnight. Subsequently, the membrane was washed with PBS-T followed by incubation with the secondary antibody conjugated with horseradish peroxidase at room temperature for 1 h. Blots were again washed with PBS-T and then developed with the ECL Plus Western Blotting Detection System (Amersham Life Science, UK).

### Isolation of lipid rafts

Lipid rafts were isolated as described previously [29]. Briefly, primary mouse macrophages were lysed in ice-cold MBS buffer (25 mM MES, pH 6.5, 150 mM NaCl, 1mM Na_3_VO_4_, 1 % Triton X-100, and protease inhibitors). Lysates were mixed with 4 ml of 40 % sucrose by mixing with 2 ml of 80 % sucrose and overlaid with 4 ml of 35 % sucrose and 4 ml of 5 % sucrose in MBS buffer. Samples were ultracentrifuged at 39,000 rpm for 18 h and fractionated into 12 subfractions.

### Quantification of cholesterol levels in lipid rafts of primary mouse macrophages

Lipid rafts were isolated as described above. Cholesterol level of lipid raft was assayed by gas-liquid chromatography as previously described [30].

### Cholesterol replenishment experiment

Primary mouse macrophages were treated with culture medium alone or medium containing SSa (3, 6, 12 μM), or MβCD (10 mM) at 37°C for 60 min. Subsequently the cells were washed with PBS and incubated with medium alone or medium containing water-soluble cholesterol (84 μg/mL) for 30min. The cells were exposed to LPS. The translocation of TLR4 to lipid rafts were analysed as mentioned above. The effects of SSa on LPS-induced cytokine production were also detected as mentioned above.

### LXR receptor gene assay

For LXR activation studies, 0.75 μg of LXRE-driven luciferase reporter vector (LXRE-tk-Luc) and 0.75 μg of β-galactosidase control vector (Promega) were used. The cells were transfected with vectors using FuGENE HP transfection reagent (Roche Applied Science, Indianapolis, IN, USA) according to the manufacturer's instructions. Six hours after transfection, cells were treated with SSa for 12 h. The β-galactosidase enzyme activity was determined using the β-galactosidase Enzyme System (Promega) according to the manufacturer' instructions. Luciferase activity was normalized by β-galactosidase activity.

### Transient transfection of siRNA against LXRα and ABCA1

The plasmid containing siRNA against LXRα and ABCA1 (si-LXRα; ON-TARGETplus SMART pool), non-targeting siRNA (si-control) and the DharmaFECT transfection reagent were purchased from Thermo Scientific Dharmacon (USA). Si-LXRα and si-control stock solutions (20 μM) were diluted with diethyl pyrocarbonate (DEPC) water to form 5 μM solutions. The DharmaFECT transfection reagent was mixed with 5μM si-LXRα or si-control, incubated for 20 min and then added to the culture medium at a final concentration of 25 μM. The cells were incubated with si-LXRα and si-control for 48 h.

### Statistical analysis

All values are expressed as mean ± SEM. Differences, between the mean values of normally distributed data assessed by ANOVA followed by Tukey-Kramer multiple comparisons test. The statistical significance was set at a level of *P* < 0.05 by convention.
